# Influence of Temperature on the Electromechanical Properties of Ionic Liquid-Doped Ionic Polymer-Metal Composite Actuators

**DOI:** 10.3390/polym9080358

**Published:** 2017-08-12

**Authors:** Abdallah Almomani, Wangyujue Hong, Wei Hong, Reza Montazami

**Affiliations:** 1 Department of Aerospace Engineering, Iowa State University, Ames, IA 50011, USA; almomani@iastate.edu (A.A.); whong@iastate.edu (W.H.); 2 Department of Mechanical Engineering, Iowa State University, Ames, IA 50011, USA; hwyj@iastate.edu; 3 Department of Materials Science and Engineering, Iowa State University, Ames, IA 50011, USA; 4 Ames Laboratory, U.S. Department of Energy, Ames, IA 50011, USA

**Keywords:** ionic electroactive polymers, IPMC, temperature, soft actuators, electromechanical actuators, soft robotics

## Abstract

Ionic polymer-metal composite (IPMC) actuators have considerable potential for a wide range of applications. Although IPMC actuators are widely studied for their electromechanical properties, most studies have been conducted at the ambient conditions. The electromechanical performance of IPMC actuators at higher temperature is still far from understood. In this study, the effect of temperature on the electromechanical behavior (the rate of deformation and curvature) and electrochemical behavior (current flow) of ionic liquid doped IPMC actuators are examined and reported. Both electromechanical and electrochemical studies were conducted in air at temperatures ranging from 25 °C to 90 °C. Electromechanically, the actuators showed lower cationic curvature with increasing temperature up to 70 °C and a slower rate of deformation with increasing temperature up to 50 °C. A faster rate of deformation was recorded at temperatures higher than 50 °C, with a maximum rate at 60 °C. The anionic response showed a lower rate of deformation and a higher anionic curvature with increasing temperatures up to 50 °C with an abrupt increase in the rate of deformation and decrease of curvature at 60 °C. In both cationic and anionic responses, actuators started to lose functionality and show unpredictable performance for temperatures greater than 60 °C, with considerable fluctuations at 70 °C. Electrochemically, the current flow across the actuators was increased gradually with increasing temperature up to 80 °C during the charging and discharging cycles. A sudden increase in current flow was recorded at 90 °C indicating a shorted circuit and actuator failure.

## 1. Introduction

Ionic polymer-metal composites (IPMCs) are polymer-based soft composites that can be designed as soft actuators and sensors. IPMC actuators have several unique properties, including low density, large bending strain, low noise, high resilience, and low operation voltage; which make their application more practical compare to many of their metal- or ceramic-based counterparts. IPMC actuators have been widely studied, experimentally, and theoretically, as artificial muscles for biomedical applications, biomimetic micro-robotics, and harsh-environment tools such as space exploration micro-grippers [1,2,3,4,5,6,7,8].

**Figure 1:**
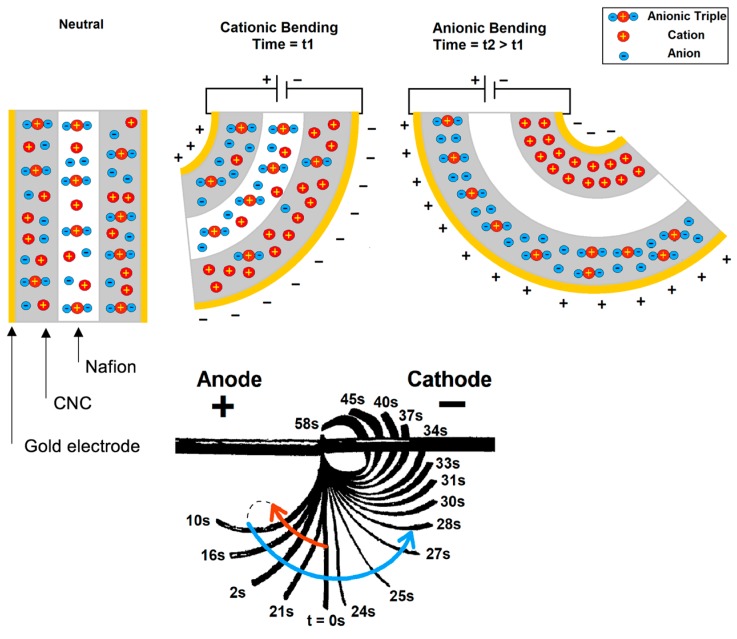
Idealistic schematic of cationic and anionic bending mechanism (top, not to scale) and overlaid sequential images of cationic (red arrow) bending followed by anionic (blue arrow) bending (bottom).

Upon fabrication, IPMCs are impregnated by optimum content [9] of ion-rich electrolyte, most often ionic liquids (ILs), as the source for mobilized ions. The operation principle of IPMC actuators is ultimately based on the accumulation of mobilized ions at the opposite electrodes in response to an externally-induced electric field. The difference between the volumes of cations and anions results in a volume imbalance and, therefore, mechanical stress, at the electrodes which, in turn, results in deformation of the IPMC structure toward the side with smaller volume. The motion of ions toward or away from each electrode is due to attractive or repulsive forces between the electrically-charged electrode and the ions, and can be reversed upon modulating the polarity of the electric field. The deformation depends on the net difference of the volumes at the electrodes. If the drift velocity of the cations and anions are significantly different, a two-step deformation is observed in which the actuator is initially bent toward one side, then toward the other side. Conventionally, the bending caused by cations (toward the anode) is called cationic bending and denoted by positive sign when that resulted by anions (toward the cathode) is called anionic and denoted by negative sign [10,11]. This change in the direction of motion is different from back-relaxation phenomenon [12,13,14]. A scheme showing the principle of actuation is presented in Figure 1.

Many factors influence dynamics of ion mobility in IPMCs including mean free path of ions which is a factor of membrane thickness as well as membrane’s chemical and physical structures [11,15,16], characteristics of ions (van der Waals volume) [11], and temperature. While characteristics of the membrane and ions are intrinsic to IPMCs and fixed, temperature is considered an external factor that can be changed for the same actuator during operation. Therefore, understanding the effect of temperature on IPMC actuators is critical in the prediction of their electromechanical behavior under different environmental conditions.

Several studies have investigated the performance of IPMC actuators as a function of temperature. Shahinpoor et al. report that IPMC actuators can function at subzero temperatures, down to −140 °C [3]. On two independent studies, Brunetto et al. and Ganley et al. describe the sensing properties and modeling of the IPMCs for a range of temperatures above the ambient temperature and humidity [17,18]. Cha et al. and Farinholt et al. considered the effect of temperature on the IPMCs’ impedance [19,20]. Benziger et al.[21] measured the elastic modulus of Nafion membrane at different temperatures and humidity and observed that under dry conditions, the elastic modulus of Nafion decreased gradually with increasing temperature (up to 60 °C), with a steep decrease at 70 °C. This behavior was mainly related to the structural changes of Nafion at high temperatures. Nafion consists of a large tetrafluoroethylene (Teflon) backbone with short perfluorovinyl ether side chains terminated with sulfonate (SO_3_^−^) end groups. In its acidic form, Nafion exists with protons (H^+^) in close vicinity of its sulfonate end groups. The most widely-accepted model for the Nafion structure was presented by Hsu and Gierke in 1982 [22]. In that model, Nafion has a cluster network structure with nano-channels that allow ion transport through the membrane. The cluster-network model suggests that Nafion contains very small (few nanometers across) hollow inverted-micelle spheres connected with nanochannels and supported by the Teflon backbone cross-links. This configuration minimizes electrostatic repulsion between the ionic groups, and the cross-links stiffen the Nafion membrane. Benziger et al. suggested that the change in mechanical properties and the steep decrease in the elastic modulus at high temperatures (>60 °C) are caused by a microstructural change in Nafion—the inverted micelles and nano-channels start to collapse due to the increase of entropy; which causes a decline in mechanical properties.

Sodaye et al. [23,24] studied the scattering parameter (S-parameter) and lifetime of dry acidic Nafion as a function of temperature and found that a larger S-value is related to a larger free volume within the Nafion structure. It was reported that, generally, the free volume increases noticeably with increasing temperature, indicating an expansion of the spherical clusters and nano-channels inside Nafion up to a threshold temperature, ~63 °C, where the free volume decreases. This decline in free volume at higher temperature is in agreement with findings reported by Benziger and colleagues, and can imply the collapse of some of the inner structures of Nafion. Such physical changes to the Nafion structure due to temperature variations, and other environmental factors such as humidity and moisture content are expected to influence its ionic and ion transport properties. Moreover, Nafion was reported to have a broad glass transition temperature within approximately 55–130 °C [25,26,27]. At the glass transition temperature, Nafion becomes more rubbery and that may also affect the structure of the membrane and the transport properties of the nano-channels in the Nafion structure and agrees with the results of Sodaye et al. and Benziger et al.

In one study, Hou et al. [28] explored the interionic associations among IL constituent ions with respect to the hydration level and reported that the electrostatic forces may result in the formation of double, triple, and quadruple ion clusters of anions and cations. When the Nafion was dry, triple anionic clusters prevailed due to the strong electrostatic forces between the charged species. Increasing water content reduced the electrostatic forces and resulted in ionic disassociation.

In this study, rate and magnitude of electromechanical response of IL-doped IPMC actuators are characterized as a function of temperature. In particular, cationic and anionic motions are independently investigated and the outcomes are integrated into a unifying model and conclusion. The dynamics of ion-cluster formation and deformation are studied utilizing electromechanical and electrochemical behavior of IPMCs in a wide range of temperatures.

## 2. Materials and Methods

### 2.1. Materials

A Nafion (sulfonated tetrafluoroethylene-based fluoropolymer-copolymer) ionomeric membrane of 25 µm thickness (NR-211, IonPower, New Castle, DE, USA) was used as received. Poly(allylamine hydrochloride) (PAH) (Sigma-Aldrich, St. Louis, MO, USA) was procured at a concentration of 10 mM and used as polycation. Functionalized gold nanoparticles (AuNPs) of ~3nm diameter, Zeta potential of ca. −40 mV, and a concentration of 20 ppm (Purest Colloids Inc, Westampton, NJ, USA) were used as anionic materials. 1-ethyl-3-methylimidazolium trifluoromethanesulfonate (EMI-Tf (molecular formula, C_7_H_11_F_3_N_2_O_3_S)) (Sigma-Aldrich, St. Louis, MO, USA) was used as received. Twenty-four carat gold leaf electrodes of 50 nm thickness (L.A. Gold Leaf Wholesaler Inc., Azusa, CA, USA) were used as the outer electrodes.

### 2.2. Sample Preparation

To fabricate the actuators, the layer-by-layer (LbL) technique [15,29] was used to deposit 20 bilayers of PAH/AuNPs and form the conductive network composites (CNCs) on both surfaces of Nafion films. The Nafion film was mounted on a glass frame using double-sided tape and an automated robot (StratoSequence 6, NanoStrata Inc., Tallahassee, FL, USA) programmed for five-minute immersions in each ionic solution followed by three one-minute rinses in DI water was used to fabricate the bilayers. After forming the CNCs, the membranes were dried in air, and cut from the glass frame to obtain free-standing IPMCs. The IPMCs were then soaked for about six hours in EMI-TF ionic liquid at 80 °C, to obtain ~30% ionic liquid intake. Ionic liquid intake (IL%) was measured as a percent ratio of weight increase to the initial weight of the membrane, using Equation (1):

(1) $$IL\left(\%\right)=\frac{W_{f}-W_{i}}{W_{i}}\times 100$$

where $W_{i}$ and $W_{f}$ are the membrane’s weights before and after IL intake, respectively. Upon impregnation with IL, gold leaf electrodes were hot-pressed onto both sides of the membrane, at 95 °C and under ~100 KN of force, for 40 s using a 25 T hydraulic hot press (MTI Corporation, Richmond, CA, USA) [10,30]. The actuators were then cut into 1 × 10 mm^2^ samples for testing.

### 2.3. Electromechanical Characterization

Electromechanical characterizations were conducted in an in-house fabricated temperature controlled chamber where a type T (−CO + CP) (Omega Engineering Inc., Norwalk, CT, USA) thermocouple paired with a thermocouple meter (Omega DP41-TC-MDS, Omega Engineering Inc., Norwalk, CT, USA) were used to measure the temperature of the chamber. The desired temperatures were obtained by manually controlling and adjusting a resistive heater. Three different actuators from the same fabricated lot were tested at each testing temperature. Before each test, enough time was allowed to obtain a uniform and stable temperature across the chamber. Actuators were mounted with a clearance of 5 cm of the heater on an in-house fabricated micro-probe station inside the chamber, and subjected to a +4 V step potential across the thickness using a function generator (Tektronix AFG 3022B, Tektronix Inc., Beavetron, OR, USA). All temperatures were within ±2 °C of the desired temperature during operation. The actuators were tested at room temperature (25 °C) and temperatures from 30 °C to 70 °C by 10-degree increments. The electromechanical response was monitored and recorded at 30 frames per second using a CCD camera. Image frames were then analyzed to obtain the actuator’s radius of curvature as a function of time *r(t)*. Time-dependent curvature *k(t)*, where *k(t) = r(t)^−1^*, was then calculated and analyzed for different temperatures.

### 2.4. Electrochemical Characterization

A VersaSTAT-4 potentiostat (Princeton Applied Research, Oak Ridge, TN, USA) was used to apply a ±4 V step potential and measure the current flow over 60-second intervals across a 1 × 1 cm^2^ Nafion membrane with 30% ionic liquid intake at room temperature (25 °C) and temperatures from 30 °C to 90 °C by 10-degree increments. The membranes were enclosed between two copper electrodes on both sides and the current density and time were then recorded for analysis.

The same device was also used to apply a 10 mV potential at frequencies ranging from 100 kHz to 0.1 Hz and to measure the samples’ electrical impedance. An equivalent RC electrical circuit with a Warburg element was previously proposed by several studies to represent the electrochemical performance across ionic liquid swollen Nafion membranes [9], from which the resistances (*R*_m_) for membranes at different temperatures can be extracted from the Nyquist plot at high frequencies (i.e., 100 kHz), where the electrochemical system exhibits almost pure resistance behavior. The ionic conductivity was then calculated for the samples at different temperatures using Equation (2):

(2) $$\sigma =\frac{t}{R_{m}A}$$

where σ is the ionic conductivity, t is the thickness of the membrane, *R*_m_ is the resistance deduced from the Nyquist plots at high frequencies, and *A* is the surface area of the membrane [31]. After that, the Arrhenius equation in its linear form Equation (3) was used to fit the experimental at different temperatures:

(3) $$\ln(\sigma )=\frac{-E_{a}}{R}(\frac{1}{T})+\ln(\sigma_{0})$$

where σ is the ionic conductivity, $\sigma_{0}$ is the maximum ionic conductivity, *E*_a_ is the activation energy, R is the gas constant, and *T* is the temperature.

## 3. Results

### 3.1. Electromechanical Response

#### 3.1.1. Cationic Curvature

**Figure 2:**
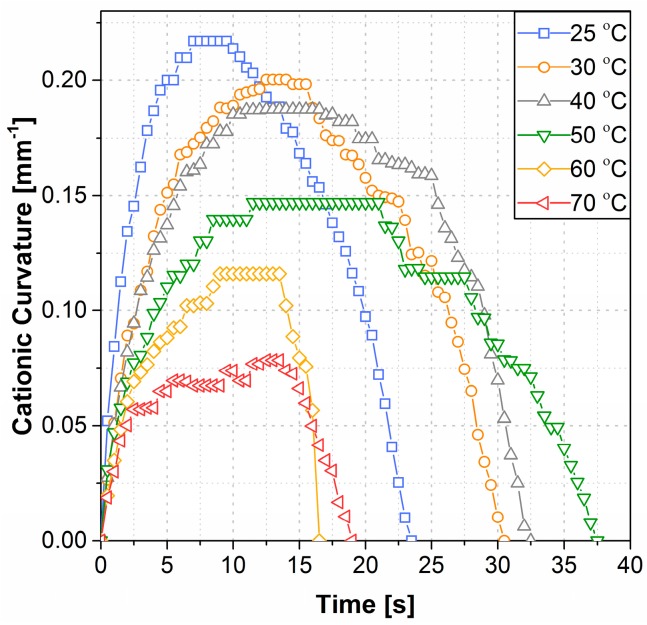
The cationic curvature at different temperatures.

**Figure 3:**
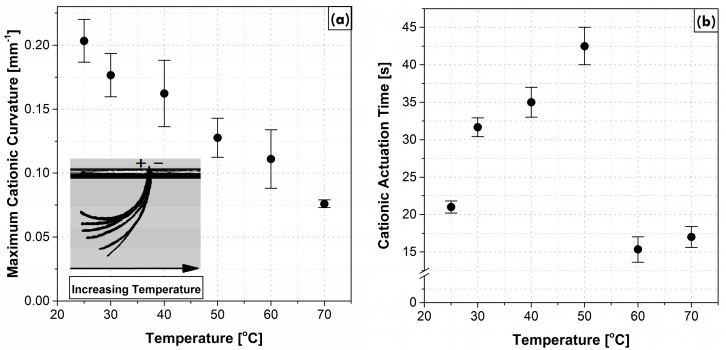
(*a*) The maximum cationic curvature; and (*b*) the cationic actuation time, at different temperatures.

The cationic curvature as a function of time at 25 °C and for temperatures from 30 °C to 70 °C by 10-degree increments is shown in Figure 2. In this figure, the curvature is considered to be cationic as cations are dominating the bending process (i.e., the bending is toward the anode, as seen in Figure 1). After applying a step voltage, the cationic curvature increased to a maximum value and then decreased to zero as time progressed. The decrease in curvature resulted from the accumulation of bigger anionic clusters at the anode [10]. Both maximum cationic curvature and the cationic actuation time (the total time for the actuator to return to the neutral position) were affected as the system temperature increased, as shown in Figure 2. The maximum cationic curvature decreased for each temperature increment from 25 °C to 70 °C, as shown in Figure 3a. Meanwhile, the cationic actuation time (from the beginning of the motion back to the idle position) first increased to a maximum at 50 °C, with a sudden decrease at 60 °C, as shown in Figure 3b. No detectable bending occurred at temperatures higher than 70 °C.

#### 3.1.2. Anionic Curvature

**Figure 4:**
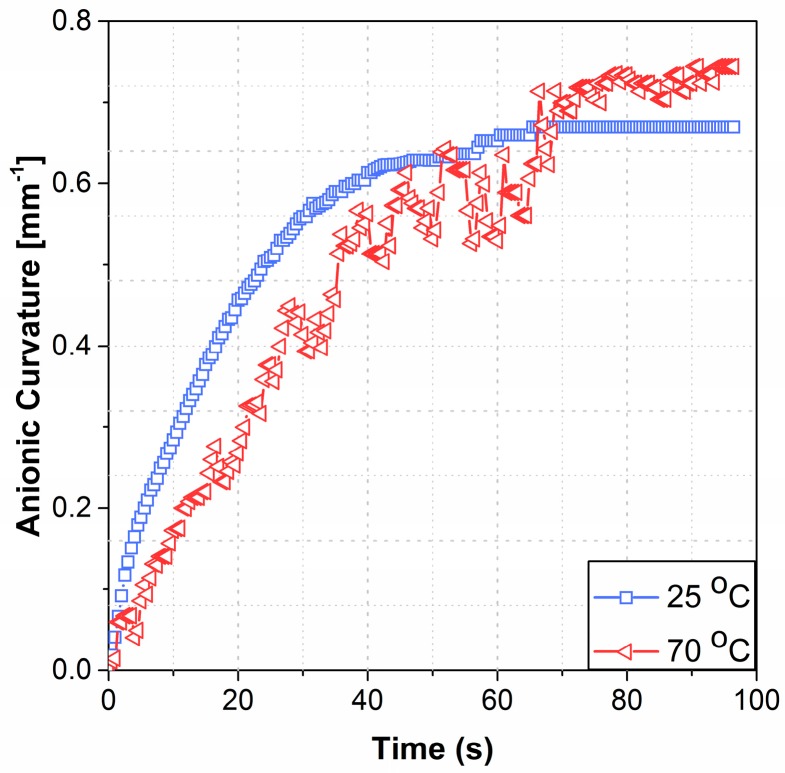
The anionic curvature at 25 °C and 70 °C, both shifted to time = zero for comparison.

**Figure 5:**
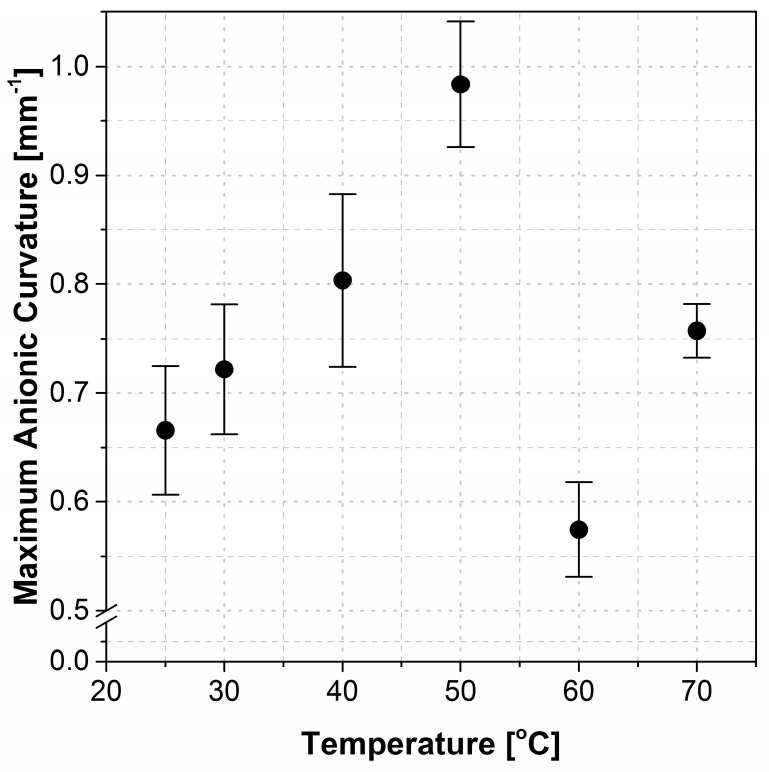
The maximum anionic curvature at different temperatures.

**Figure 6:**
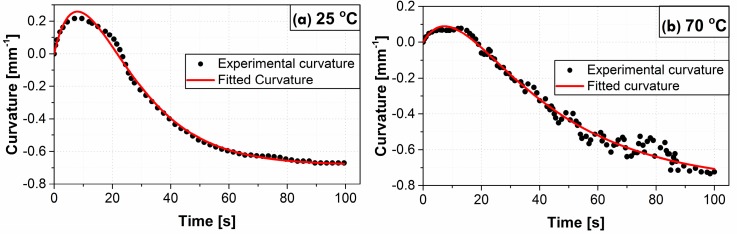
Experimental and fitted time dependent curvature at (*a*) at 25 °C, and (*b*) 70 °C.

Anionic curvature increased to a steady-state maximum value at each tested temperature from 25 °C through 70 °C. The anionic curvature as a function of time exhibited smooth deformation for temperatures ranging from 25 °C to 50 °C (see Figure 4 for a representative example). However, the actuator deformation fluctuated noticeably past 50 °C and up to 70 °C. Anionic curvatures for 25 °C (smooth) and 70 °C (fluctuating) are shown in Figure 4. The two curves were shifted to the zero time to be compared side-by-side, see Figure 6 for the full actuation cycle data. The maximum anionic curvatures for actuators tested at 25 °C and for temperatures from 30 °C to 70 °C by an increment of 10 °C are shown in Figure 5. The maximum anionic curvature increased to a peak value as the temperature increased up to 50 °C and dropped abruptly after that. At temperatures above 70 °C, actuators only showed unpredictable oscillation at the idle position with no clear cationic or anionic curvatures.

#### 3.1.3. The Time Constant for Anionic and Cationic Curvatures

A two-part first-order system model was used to fit the rate of deformation data for both cationic and anionic motions. In this model, both cations and anions are mobilized once the voltage is applied. Cationic and anionic curvatures were considered to have positive and negative values, respectively, to distinguish their opposing directions of motion. The model was produced using Equation (4) with a negative sign for the anionic motion [15,32]:

(4) $$k(t)=k_{cat}\left(1-\exp(\frac{-t}{\tau_{cat}})\right)-k_{an}\left(1-\exp(\frac{-t}{\tau_{an}})\right)$$

where *k(t)* is the net curvature as a function of time, *k*_cat_ and *k*_an_ are the cationic and anionic coefficients denoting the maximum value of each, *t* is the time, and $\tau_{cat}$ and $\tau_{an}$ are the cationic and anionic time constants, respectively. Experimental data fitted with analytical curves for 25 °C and 70 °C are shown in Figure 6.

**Figure 7:**
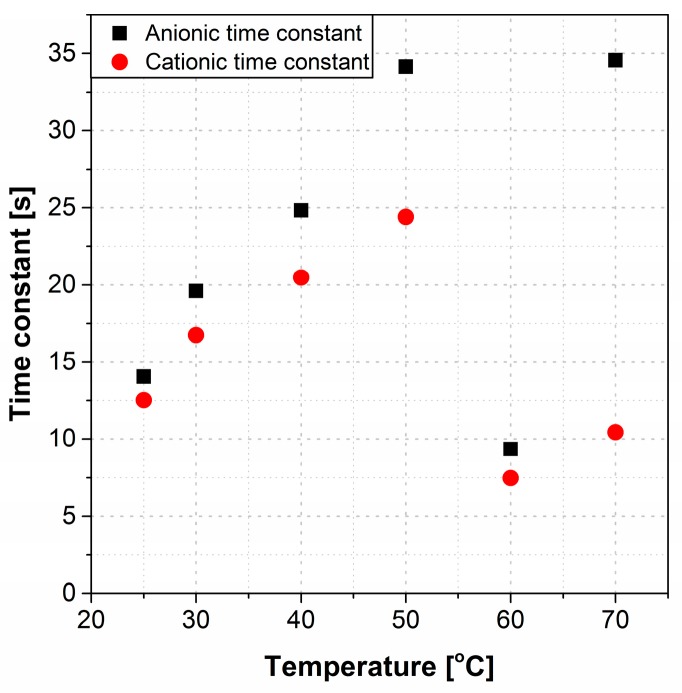
The time constant of cationic and anionic curvatures at different temperatures.

The time constants at different temperatures are shown in Figure 7. The actuators had a lower rate of deformation (both $\tau_{cat}$ and $\tau_{an}$ increased) with increasing temperatures up to 50 °C. A sudden drop in $\tau_{cat}$ and $\tau_{an}$ values were observed at 60 °C. At 70 °C the rate of deformation was small, yet associated with considerable fluctuations in motion.

### 3.2. Electrochemical Characterization

**Figure 8:**
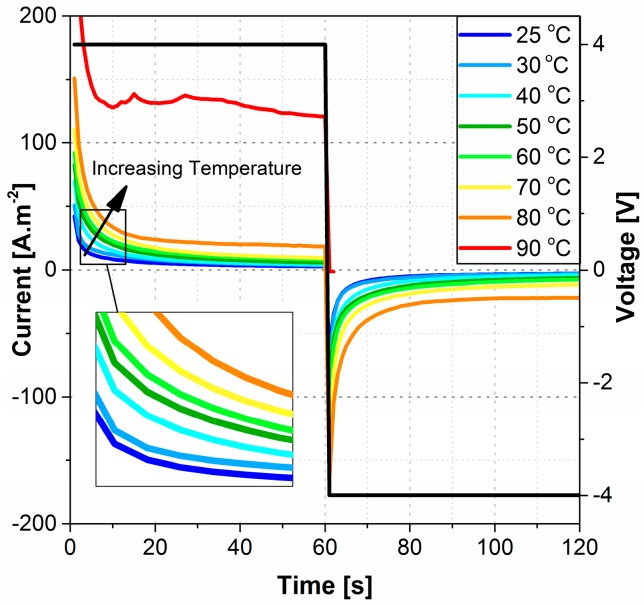
The current flow after applying 4 V across a 1 × 1 cm^2^ Nafion membrane with 30% EMI-TF ionic liquid at different temperatures. The leftmost gray line is the current flow at room temperature; each colored line above it shows an increase in temperature from 30 °C to 90 °C.

Presented in Figure 8 is the time dependent current flow measured at different temperatures. Despite the lack of electromechanical response at temperatures higher than 70 °C, electrochemical behavior of the actuators followed a constant pattern up to 80 °C, and exhibited a jump along with a relatively flat (time independent) high current flow at 90 °C indicating physical and/or chemical degradation of the actuator. The system then completely failed after about 60 s at 90 °C when the current flow dropped to zero.

**Figure 9:**
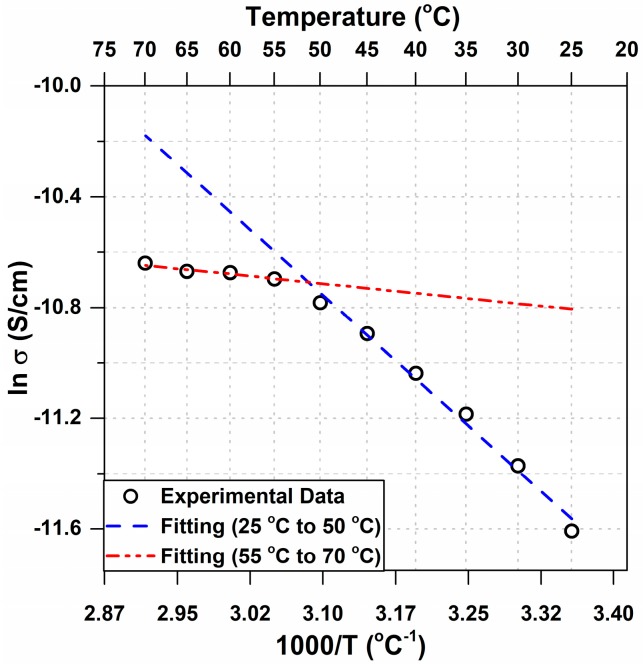
Arrhenius conductivity fitting for temperatures from 25 °C to 70 °C.

Figure 9 shows the ionic conductivity across the membranes calculated at different temperatures. The results showed an enhanced conductivity at higher temperatures. Moreover, a different conductivity behavior was noticed for the temperature range between 25 °C and 50 °C than the temperature range between 55 °C and 70 °C after fitting the conductivity data with the Arrhenius equation (Equation (3)).

## 4. Discussion

Experimental data confirmed two distinct deformation patterns for cationic and anionic curvatures, suggesting that each is differently affected by temperature. The magnitude of the cationic curvature exhibited an inversely proportional dependence on temperature as the temperature was increased from 25 °C to 70 °C (Figure 3a); while this correlation for anionic curvature was a direct correlation up to 50 °C followed by a sharp drop at 60 °C and increase at 70 °C (Figure 5). The irregular behavior of the anionic curvature was also observed for time constants of both cationic and anionic curvatures (Figure 7).

It is speculated that the irregular behavior of the IPMCs at higher temperatures is due to two main factors: (1) changes in Nafion’s nano-structure, and (2) changes in IL composition and transportation through Nafion.

Changes in Nafion’s nano-structure has been subject to a number of studies, as described in the introduction section, and while it partially explains temperature dependent behavior of IPMC actuators, it fails to explain the distinct behaviors of cationic and anionic curvatures. It is speculated that changes in IL composition and transportation at different temperatures have a strong contributing factor to such distinct behaviors and can be used to explain the temperature-dependent behavior of IPMC actuators.

Deformation of IPMC actuators is a result of ion mobility, in case of this study EMI^+^ and Tf^−^. Previous studies have indicated presence of Tf^−^-EMI^+^-Tf^−^ anionic clusters in EMI-Tf ionic liquid; which are considerably more massive than EMI^+^ cations. Increasing the number of anionic clusters will enhance anionic curvature, considering the more significant difference in van der Waals volume of the cluster compared to that of EMI^+^, and the fact that for each cluster one cation is contributing to anionic curvature rather than, naturally, cationic curvature. However, the drifting velocity of such ionic clusters is lower due to the increased mass for the net one elementary charge. The drifting velocity *V_d_* is reversibly related to the ion mass as shown in Equation (5) [33]:

(5) $$V_{d}=2\sqrt{\frac{KT}{3m}}$$

where *K* is the Boltzmann constant, *T* is absolute temperature, and *m* is the mass of the ion.

The magnitude of the maximum cationic curvature (Figure 3a), and the cationic and anionic rates of deformation (Figure 7), are both consequent to the net difference in magnitudes of cations and anions (including anionic clusters) drifting velocities that are vectors pointing at opposite directions or the amount of the moving ions. A larger net difference in velocities or a lower amount of moving anions/anionic clusters results in a higher maximum cationic curvature, as well as higher cationic and ionic rates of deformation, and vice versa. The magnitude of the maximum anionic curvature (Figure 5), on the other hand, solely depends on the net volume difference of the accumulated ions at the two electrodes which, in turn, depends on the abundance of anionic clusters accumulated at the anode.

The maximum cationic curvature decreases with increasing temperature from 25 °C through 70 °C. This indicates a lower net difference between the cations and anions/anionic clusters drifting velocities or a higher number of anions/anionic clusters are being drifted and canceling the cationic motion. Figure 7 shows higher time constants for both cationic and anionic motions with increasing temperature. The higher time constant may conclude a slower drifting velocity of the charged species and lower current density. Instead, the current flow across the Nafion membranes increases with increasing temperature as shown in Figure 8. As cations are smaller in size and less massive than the anions/anionic clusters, they assumed to move freely at all temperatures. Thus, the number of moving cations is assumed to be the same or differ slightly at elevated temperatures. On the other hand, some of the larger and more massive anionic clusters will be trapped inside the Nafion’s nano-channels at low temperatures. Increasing temperature will expand Nafion’s nano-channels and increase the kinetic energy of the clusters, which increases the number of the moving anions/anionic clusters with a lower drifting velocity will explain the lower maximum cationic curvature and lower rate of deformations with increasing temperature up to 50 °C. To illustrate this, the current flow across the membrane can be modeled by Equation (6):

(6) $$I\propto -C_{0}\exp(\frac{-E_{a}}{RT})[1-\exp(\frac{-t}{\tau (T)})]+C_{1}$$

where *I* is the current flow across the membrane, C_0_ is a constant related to the concentration of the IL, *E*_a_ is the activation energy, R is the gas constant, *T* is the temperature, *t* is time, *𝜏* (*T*) is the time constant as a function of temperature, and *C_1_* is a constant to represent the steady state current flow. The equation shows that a higher current could be achieved with increasing temperature for the same initial IL concentration even for a higher time constant. Moreover, protons (H^+^) have a great effect on the current flow. Protons are significantly smaller compared to EMI^+^ and Tf^−^ ions, making it easier for them to drift across the Nafion membrane. Due to their size, increasing the protons’ kinetic energy will result in a greater current flow. This is true even at higher temperatures that break the inner nano-structure of Nafion. At 90 °C, the high current flow indicates a short circuit and actuator failure.

Anionic curvature increases with increasing temperature up to 50 °C followed by a sudden decrease in curvature which is concurrent with an increase in the rate of deformation (decreasing time constant) occurred at 60 °C. The increase in anionic curvature can be explained by the larger number of anionic clusters accumulated at the anode due to the increased temperature. In addition to the ions’ greater kinetic energy, ion transport through Nafion is also expected to be facilitated by the expansion of the nano-structure of Nafion due to the increasing temperatures up to 50 °C, before a physical change in both the IL composition and the Nafion nano-structure at temperatures above 50 °C occur.

At 60 °C, there was an increase in both cationic and anionic rates of deformations and a steep drop in the anionic curvature. If ions become smaller (ion clusters dispersion into single ions), lower curvature and a higher rate of deformation result. It is speculated that at high temperatures (>50 °C), the kinetic energy of some the ions forming the ion clusters will be higher than the potential energy of the clusters. This will cause a dispersal of some of the clusters into single ions and increase the entropy of the system. Thus, at high temperature, ionic dispersal will result in a lower number of the anionic clusters to exist and decrease the net anionic curvature. The high energy and higher number of small single ions will result in a higher ion transport rate and a higher rate of deformation (time constant decreases). Ionic dispersal hypothesis can be supported by the conductivity results at different temperatures shown in Figure 9. The slope of the linear fitted line for different temperature ranges represents the activation energy needed for ion transportation. The activation energy is lower for temperatures >50 °C which concludes a smaller ionic species. Smaller ionic species shall result in an even higher conductivity (the conductivity at >50 °C should lie above the single dashed blue line in Figure 9). Instead, the conductivity at >50 °C is lower than the previous range trend. This might be due to the change and the breakage of some nano-channels in Nafion structure. Upon dispersion of ion clusters, both cationic and anionic curvatures again follow the same trend as that observed for lower temperatures, i.e., the decreasing rate of deformation as the temperature increases to 70 °C, with a steep decrease for the anionic curvature (Figure 7). Benziger et al. demonstrated that, in dry conditions, the elastic modulus shows a steep decrease at temperatures between 70 °C and 100 °C [21]. The sudden decrease in modulus is related to the increase of entropy at high temperatures. This extra entropy randomly disperses the ionic groups in Nafion and breaks some of the inner structures and nano-channels. Breaking these structures also affects the ions’ transport across the membrane. Majsztrik et al. reported the same results in their study about the effects of temperature and hydration on tensile creep viscoelastic response of Nafion [34]. Thus, breaking of some of the inner channels and clusters at higher temperatures can affect and limit the ion transport, and explain both the decrease in the rate of deformation and the decrease in the actuation curvature. The cluster breakage has more of an effect on the anionic rate of deformation, which results from the slower movement of the larger anionic clusters. That explains the greater difference in the rate of deformation at 70 °C (Figure 7).

## 5. Conclusions

In summary, this work investigates the effect of temperature on the electromechanical performance of IL-doped IPMC actuators. Cationic and anionic curvatures exhibited two distinct behaviors as a function of increasing temperature. Considering changes in the kinetic energy of ions as a function of temperature, ion mobility using Arrhenius relation, and physical changes of Nafion’s nano-structure, it is concluded that the complex electromechanical behaviors of IL-doped IPMC actuators at higher temperatures can be explained by changes in IL structure, i.e., dispersion of ion clusters in the IL and the number of the drifted ions at higher temperatures. It is suggested that the kinetic energy of the mobilized ions is higher than the potential energy of the ionic clusters at higher temperatures, which results in the dispersal of ionic clusters.
